# Correction to Retraction of: Kidney Injury Molecule-1 Is Up-Regulated in Renal Epithelial Cells in Response to Oxalate *In Vitro* and in Renal Tissues in Response to Hyperoxaluria *In Vivo*

**DOI:** 10.1371/journal.pone.0236263

**Published:** 2020-07-13

**Authors:** The PLOS ONE Editors

## Notice of Republication

The retraction notice [1] for this article [2] was republished on June 25, 2020 to correct an error that wrongly named the Louisiana State University Shreveport as one of the institutes involved in the joint investigation into the concerns with Fig 3B. The institutes involved in this joint investigation are The University of Colorado and Louisiana State University Health Sciences Center at Shreveport. The publisher apologizes for this error. Please download this retraction notice again to view the correct version. The originally published notice and the republished, corrected notice are provided here in S1 and S2 Files respectively, for reference.

## Supporting information

S1 File Originally published, uncorrected retraction notice. (PDF)

S2 File Republished, corrected retraction notice. (PDF)
