# Supplementary material for: Correction to Retraction of: Kidney Injury Molecule-1 Is Up-Regulated in Renal Epithelial Cells in Response to Oxalate In Vitro and in Renal Tissues in Response to Hyperoxaluria In Vivo
Source: PLoS One. 2020 Jul 13;15(7):e0236263. doi: 10.1371/journal.pone.0236263 (PMC7357772; doi:10.1371/journal.pone.0236263)
Supplement: S2 File — (PDF) [file pone.0236263.s002.pdf]

PLOS ONE

RETRACTION

Retraction: Kidney Injury Molecule-1 Is Up-Regulated in Renal Epithelial Cells in Response to Oxalate *In Vitro* and in Renal Tissues in Response to Hyperoxaluria *In Vivo*

The *PLOS ONE* Editors

Following the publication of this article [1], concerns were raised regarding Fig 3:

- Fig3B, first Hyper-oxaluric panel, there is an appearance of a vertical discontinuity between the first and second lane.
- Fig 3B, third Hyper-oxaluric panel, there is an appearance of vertical discontinuities on either side of the third lane.

The authors provided underlying data that verified some but not all of the bands presented in Fig 3B. The underlying data for the remaining bands are no longer available. The corresponding author said that the underlying data are no longer available because the authors changed institutions.

The University of Colorado and the Louisiana State University Health Sciences Center at Shreveport jointly investigated concerns regarding Fig 3B, but were unable to clarify the issues regarding this figure and how the experiment was conducted based on available laboratory records. The authors offered repeat experiment data for Fig 3B, but these repeat data do not resolve the concerns summarized above.

In light of the above concerns that call into question the integrity of results reported in Fig 3B, the *PLOS ONE* Editors retract this article.

LK and HKK agreed with the retraction. SK and RBM either did not respond directly or could not be reached. HKK stands behind the overall conclusions presented in the article.

Check for updates

Reference

1. Khandrika L, Koul S, Meacham RB, Koul HK (2012) Kidney Injury Molecule-1 Is Up-Regulated in Renal Epithelial Cells in Response to Oxalate *In Vitro* and in Renal Tissues in Response to Hyperoxaluria *In Vivo*. PLoS ONE 7(9): e44174. https://doi.org/10.1371/journal.pone.0044174 PMID: 22984472

OPEN ACCESS

Citation: The *PLOS ONE* Editors (2020) Retraction: Kidney Injury Molecule-1 Is Up-Regulated in Renal Epithelial Cells in Response to Oxalate *In Vitro* and in Renal Tissues in Response to Hyperoxaluria *In Vivo*. PLoS ONE 15(6): e0234862. https://doi.org/10.1371/journal.pone.0234862

Published: June 11, 2020

Copyright: © 2020 The PLOS ONE Editors. This is an open access article distributed under the terms of the Creative Commons Attribution License, which permits unrestricted use, distribution, and reproduction in any medium, provided the original author and source are credited.
